# Teen mothers’ experiences with youth-friendly health services in Eastern Cape, South Africa

**DOI:** 10.4102/jphia.v16i1.1151

**Published:** 2025-05-30

**Authors:** Zikhona S. Ngqola, Carine Prinsloo

**Affiliations:** 1 Department of Health Studies, College of Health Studies, University of South Africa, Pretoria, South Africa

**Keywords:** adolescent, adolescent youth-friendly services, experiences, primary healthcare facilities, primary healthcare providers, teenage mothers, youth-friendly policies

## Abstract

**Background:**

Teenage mothers face significant challenges, including social stigma, a lack of support and inadequate healthcare services, which adversely affect their well-being and parenting capacities. These challenges are compounded by a lack of youth-friendly healthcare services tailored to their unique needs.

**Aim:**

This study aimed to explore the lived experiences of teenage mothers regarding the youth-friendliness of support services in primary healthcare facilities in the Enoch Mgijima district in the Eastern Cape.

**Setting:**

The study was conducted in primary healthcare facilities in the Enoch Mgijima district, focusing on teenage mothers aged 10–19.

**Methods:**

An exploratory qualitative research design was employed. Data were collected through in-depth interviews with 10 teenage mothers selected using convenience and snowball sampling techniques. Data analysis followed Braun and Clarke’s reflective thematic analysis framework.

**Results:**

From the data, four main themes and ten subthemes were identified. The findings emphasise the need for more inclusive, confidential and accessible healthcare services tailored to the unique needs of teenage mothers, particularly those balancing school and caregiving or living in remote locations.

**Conclusion:**

Enhancing healthcare worker training, promoting comprehensive sexual and reproductive health education, and tailoring services to meet the needs of teenage mothers are essential to improving health outcomes.

**Contribution:**

The study highlights gaps in youth-friendly healthcare services and proposes actionable recommendations to foster supportive, inclusive and accessible healthcare for teenage mothers in rural South Africa.

## Introduction and background

Teenage mothers, defined as adolescents aged 10–19 years who give birth,^1^ face immense challenges that amplify the already complex transition from childhood to adulthood. Despite adolescence often being considered a physically healthy phase, teenage mothers face profound social, economic and health-related challenges. These include societal stigma, the loss of social connections and financial dependence, frequently leaving them reliant on unsupportive families or coping with neglect in the absence of robust support systems.^2^ The lack of paternal involvement further isolates these young mothers, increasing their vulnerability to mental health issues such as posttraumatic stress disorder.^2^

These difficulties are often rooted in systemic inequities. Many teenage mothers come from disadvantaged backgrounds characterised by family instability, unsafe environments and limited access to resources, leaving them susceptible to childhood traumas, including sexual abuse, which are linked to early pregnancies.^3^ These intersecting challenges highlight an urgent need for healthcare systems to deliver accessible, adolescent-centred services that are respectful, high-quality and well-coordinated, as recommended by the World Health Organization’s (WHO) standards for teenage-friendly care.^4^ However, the healthcare system’s insufficient focus on the unique needs of teenagers remains a significant barrier, restricting access to effective support. Bridging these gaps is essential to enhancing the welfare of teenage mothers and reducing the long-term, intergenerational impacts of their struggles.

Teenage pregnancy is a global issue with significant economic, social and health consequences. Pregnant teenagers face increased risks of conditions such as eclampsia, puerperal endometritis and systemic infections,^5^ while their infants are more likely to experience low birth weight, preterm birth and severe neonatal complications.^6^ The challenges of teenage pregnancy are exacerbated by the lack of essential knowledge, skills and resources needed for early parenthood, compounding the already stressful development of adolescents.^7^

In 2021, sub-Saharan Africa recorded the highest number of births among adolescents aged 15–19 years.^8^ Primary healthcare (PHC) is the first point of contact for many teenage mothers, encompassing maternal health services such as antenatal and postnatal care. These young mothers need tailored healthcare, education and supportive programmes to help them navigate parenthood effectively.^7^ The WHO recommends collaborative efforts to raise awareness of adolescent pregnancy, establish evidence-based policies and develop programmatic tools to improve access to quality maternal care for pregnant teenagers.^8^

Essential healthcare services for teenagers should include educational materials on sexual and reproductive health (SRH), information on contraceptive options, pregnancy signs, adoption processes, child support grants and nonjudgemental care from providers.^9^ Developed countries emphasise the integration of comprehensive support services through interdisciplinary approaches to enhance mental health and overall well-being. Family centres in the Netherlands and Norway provide universal, preventive and specialised services designed to support young people and their families.^10^ Joronen^10^ further mentioned that school-based services in countries such as the Netherlands, England and Finland play a crucial role in fostering student engagement and addressing psychosocial challenges.

In contrast, teenagers in developing countries encounter numerous barriers, including stigma, sociocultural norms, healthcare providers’ attitudes, a lack of confidentiality, high transportation costs and limited knowledge of available services.^11^ For example, pregnant teenagers in Uganda face cultural and systemic inadequacies in youth services, necessitating regulatory reforms.^12^ Youth-friendly services implemented in Zambia have increased teenage satisfaction and professional support for SRH services.^13^

South Africa has introduced the National Adolescent and Youth Policy to guide healthcare providers and stakeholders in improving adolescent health through an integrated approach. This policy promotes healthy lifestyles, reduces risks and prioritises early detection and intervention.^14^ Despite these efforts, rural areas still face challenges, including barriers to healthcare access and poor service delivery, highlighting the need for continued focus on improving teenage-specific health services.^13^

Adolescent Youth Friendly Services (AYFS) were developed to promote health strategies and interventions addressing SRH. These services aim to create a supportive and safe environment for teenagers, transforming the healthcare system to ensure AYFS are accessible. They focus on providing counselling and skills development to empower teenage mothers with life options.^13^

An assessment of AYFS in healthcare facilities in Gauteng and North West provinces of South Africa revealed that they failed to meet youth-friendly service criteria. Specific measures such as supportive management, policy implementation and information and communication processes scored poorly.^13^ Similarly, a study in Ugu, KwaZulu-Natal, indicated the absence of healthcare services tailored to the unique needs of teenage mothers.^7^ Overall, studies on AYFS in South African PHC facilities report a significant shortage of teenage-specific services, particularly in rural areas, where low utilisation rates and inadequate knowledge of sexual and reproductive healthcare services are prevalent.^7,13^

While several studies have explored AYFS for teenage mothers at public healthcare facilities, none, to the researcher’s knowledge, have been conducted in the Eastern Cape. As a professional nurse working in a PHC facility, the researcher has observed poor attendance by teenage mothers and a general lack of awareness about SRH services. The study aimed to explore and understand the lived experiences of teenage mothers regarding the youth-friendliness of support services available to them at a PHC facility in the Enoch Mgijima district in the Eastern Cape.

## Research methods and design

### Study design and site

The researcher adopted an exploratory qualitative research design to gain deeper insights and clarity into the experiences of teenage mothers utilising youth-friendly support services in PHC facilities in Molteno, a small rural town in the Enoch Mgijima district in the Eastern Cape. These facilities provide reproductive services, immunisation, basic antenatal care, tuberculosis and HIV management and care for minor ailments and chronic medication. According to Van Niekerk et al.,^15^ the Eastern Cape has the highest proportion of teen mothers.

### Sampling and recruitment

The target population for this study comprised teenage mothers in the Enoch Mgijima district in the Eastern Cape. The study included all teenage mothers aged 10–19 years residing in Molteno, including those who had visited the clinic as well as those not currently using the clinic. Teenage mothers who had given their babies up for adoption or whose babies were being cared for by a family member and those who had never visited the clinic were excluded from the study. Participants were recruited using nonprobability convenience sampling and snowball sampling methods. Convenience sampling was used to select teenage mothers who had previously utilised the three PHC services, while snowball sampling was employed to identify teenage mothers who were not currently accessing these services. In total, 10 participants were included in the study, with seven selected through convenient sampling and three through snowball sampling.

At the three PHC facilities, teenage mothers attending postnatal visits were approached by a designated gatekeeper, an administrative clerk at the reception, who provided them with an information letter about the study. Interested participants left their contact details with the gatekeeper, allowing the researcher to follow up directly. The researcher then contacted willing participants to arrange appointments with them and their guardians. During these meetings, the researcher provided a verbal explanation of the study, answered any questions and ensured informed consent and assent were obtained. Guardians signed the consent forms, while minor participants signed assent forms to confirm their agreement to participate.

In addition, teenage mothers who participated in the study were asked whether they knew other teenage mothers not utilising PHC services. These individuals were invited to contact the primary researcher if they were interested in participating. For those who expressed willingness, the researcher scheduled appointments, provided them and their guardians with information letters, provided verbal explanations of the study and collected the appropriate signed consent or assent forms. This recruitment approach ensured a diverse sample of participants, capturing both users and nonusers of PHC services.

### Data collection

#### Researcher’s role

In this study, the researcher was the primary investigator responsible for conducting interviews, collecting data and analysing findings. Although the researcher is a professional nurse working in the antenatal section of the PHC facility, the researcher was not the designated healthcare provider responsible for the antenatal care of any participants. While the researcher could have had professional contact with participants when they visited the antenatal clinic, the researcher’s role in the study was strictly separate from their clinical responsibilities. The study design ensured that interactions during the research process were independent of the routine healthcare provided.

#### In-depth interviews

In-depth interviews were used as the primary data collection method. A semistructured interview guide ensured consistency and allowed flexibility to explore emerging themes. The interviews were conducted in a private consultation room during 2022, each lasting approximately 20 min to 30 min. Because of the coronavirus pandemic, the WHO (2020)^16^ guidelines and lockdown regulations were implemented during the interviews. The researcher began by introducing herself and explaining the study’s purpose. Participants were assured that their involvement in the study would remain confidential. Their names were not used to protect their identities, and each participant was assigned a pseudonym, such as Person A, which was used throughout the study. No names were included during the transcription process to maintain privacy and anonymity. Participants were also informed of their right to ask questions or withdraw from the study at any time. The researcher adopted a friendly and approachable demeanour to foster a comfortable and open environment.

The interviews were conducted in English to facilitate clear communication without the need for interpretation. Participants were encouraged to share their experiences in their own words, and probing questions were used to gather comprehensive information. By the 10th interview, no new insights had emerged, and the depth of understanding was consistent across participants.

### Data analysis

Braun and Clarke’s reflective thematic analysis was employed for data analysis, utilising their six-phase process to systematically guide the researcher in identifying and examining key patterns in the data.^17^ This approach facilitated a comprehensive analysis by focusing on the significant aspects of thematic interpretation. The authors analysed the data individually, followed by a consensus meeting to refine and finalise the themes. From the data, four main themes and 10 subthemes were identified, as seen in Table 1.

**TABLE 1:** Themes and subthemes.

Themes	Subthemes
1. Access to public healthcare services	1.1 Utilisation of healthcare services 1.2 Knowledge of available services 1.3 Challenges accessing public healthcare services
2. Support for teenage mothers	2.1 Family and partner support 2.2 Healthcare workers support 2.3 Public health services support
3. Healthcare workers’ working ethics	3.1 Judgemental attitude and perceptions of healthcare workers 3.2 Negative behaviour and practices of healthcare workers
4. Training and teaching	4.1 Training of healthcare workers 4.2 Teaching of teenage mothers

*Source:* Ngqola Z. Lived Experiences of Teenage Mothers Regarding Youth-Friendly Support Services in Primary Healthcare Facilities in the Eastern Cape [MPH Dissertation]. University of South Africa.

### Ethical considerations

Ethical clearance to conduct this study was obtained from the University of South Africa College of Human Sciences Research Ethics Committee (No. 63332736_CREC_CHS_2021) and the Province of the Eastern Cape Health (No. EC_202111_003). Ethical principles related to human rights – including anonymity, confidentiality, privacy, beneficence, justice, and autonomy – were strictly upheld throughout the study. Permission to conduct the research was obtained from the relevant institutional authorities, ensuring compliance with institutional and ethical guidelines. Informed consent was obtained from the participants’ guardians, and assent was secured from the participants themselves after explaining the voluntary nature of their involvement and the purpose of the study. All participants were assured of their right to withdraw at any stage without any consequence, and measures were taken to protect their identity and the confidentiality of the information provided.

## Results

The study’s key findings are structured around the identified themes and subthemes outlined in Table 1, providing the perceptions of teenage mothers’ experiences with youth-friendly healthcare services.

### Theme 1: Access to public healthcare services

Access to healthcare services plays a crucial role in promoting the well-being of teenagers, as preventative measures can identify risky behaviours, encourage healthy habits and improve overall health outcomes.^18^ However, the findings revealed mixed experiences among teenage mothers regarding the utilisation, knowledge and accessibility of these services.

#### Subtheme 1.1: Utilisation of healthcare services

Teenage mothers reported frequent use of PHC services for child immunisations, family planning and personal postnatal care. Services such as antenatal care, vaccinations, family planning and weight monitoring were commonly accessed, highlighting the functional availability of essential services. Statements such as:

‘I do go to the clinic for my family planning and baby’s immunisation dates.’ (Person A, 16 years, Grade 9)

‘Every month for the baby visit and also for my visits.’ (Person D, 15 years, Grade 8)

Reflect their reliance on these services. However, underutilisation of other critical services, such as cervical screening and education on sexually transmitted infections (STIs), was noticed, with some participants expressing dissatisfaction with the treatment received. For instance:

‘I don’t like the services because of the treatment that we get there.’ (Person F, 18 years, Grade 11)

‘But I don’t like going to the clinic.’ (Person B, 16 years, Grade 9)

#### Subtheme 1.2: Knowledge of available services

The findings revealed a significant knowledge gap among teenage mothers regarding the full range of services available to them. Most participants were aware of contraception and child immunisation services but lacked comprehensive SRH education. This was partly attributed to cultural taboos and limited parental engagement. Participants expressed their lack of awareness with statements such as:

‘I do not know what type of services I am supposed to get besides immunisation for my daughter.’ (Person F, 17 years, Grade 11)

‘I only know about contraception there is not much that I know of.’ (Person C, 14 years, Grade 7)

‘We are not well informed regarding the services that we are to receive.’ (Person H, 17 years, Grade 10)

#### Subtheme 1.3: Challenges accessing public healthcare services

Several barriers to accessing healthcare services were identified. Teenage mothers cited embarrassment, a lack of confidentiality, judgemental attitudes from healthcare workers and limited accommodation for school-going teenagers. Participants described difficulties balancing school and clinic visits, with some missing school or clinic appointments altogether. For example:

‘I was chased away in the clinic for bringing the baby after school.’ (Person F, 18 years, Grade 11)

Privacy concerns were also prominent, as participants felt exposed in small community settings where clinic staff were known to their families, leading to a reluctance to seek care. One participant stated:

‘Our community is too small … we are scared of using the services because our problems will be known by our parents.’ (Person E, 17 years, Grade 11)

Geographic barriers were noted, particularly for those living in remote areas, as illustrated by:

‘As a person that stays on the farm … we use the services when the mobile clinic comes.’ (Person I, 16 years, left school at Grade 10)

### Theme 2: Support for teenage mothers

The findings underline the importance of support systems for teenage mothers, particularly from family, partners, healthcare workers and public health services. These support structures play a critical role in mitigating the challenges faced by teenage mothers and enhancing their overall well-being.

#### Subtheme 2.1: Family and partner support

Participants highlighted that family support, particularly from mothers, was instrumental in managing their responsibilities. Many teenage mothers relied on their mothers to care for their babies while attending school or engaging in other activities. Statements such as:

‘My mother helps me with the baby.’ (Person B, 16 years, Grade 9)

‘My mother assists me with taking care of the baby because it gets overwhelming at times when I have to do it alone and also be busy with schoolwork as well.’ (Person E, 17 years, Grade 11)

Exemplify the significance of maternal support. Some participants also reported receiving help from other family members, such as aunts. However, not all participants experienced such support, with a few observing that the lack of family assistance forced them to drop out of school, as seen in the case of one participant whose mother refused to care for the baby until the child was 3 years old.

Partner support was less consistent. Most participants reported no involvement or support from the fathers of their children, with some fathers abandoning them on learning of the pregnancy. Participants opined the following:

‘Because the father of my child left me and I no longer communicate with him.’ (Person A, 16 years, Grade 9)

‘The father of my child left me once I told him that I was pregnant.’ (Person E, 17 years, Grade 11)

One participant indicated some level of support from her partner:

‘I did not receive any support from my family so that is why I decided to drop out, my boyfriend is supportive I can say because I stay with him so he doesn’t have a choice to assist me.’ (Person I, 16 years, left school in Grade 10)

And one participant mentioned that she didn’t receive any support from her family:

‘I dropped out during grade 11. I was thinking that I will return after delivering but my mother refused to take care of my child until she is 3 years old.’ (Person G, 19 years, left school in Grade 11)

#### Subtheme 2.2: Healthcare worker support

Healthcare workers were identified as a crucial source of support, particularly for providing counselling and guidance on baby care. Participants expressed appreciation for healthcare workers who were attentive and understanding, with one noting:

‘The sisters help me a lot on how to care for the baby after delivery, showing us how to breastfeed, monitoring the baby’s weight, and also for immunizations.’ (Person B, 16 years, Grade 9)

However, several participants expressed dissatisfaction with judgemental attitudes and a lack of empathy from some healthcare providers, emphasising the need for more youth-friendly approaches:

‘I would appreciate if we are not neglected and judged when we go to the health facilities, especially by the nurses.’ (Person A, 16 years, Grade 9)

#### Subtheme 2.3: Public health services support

Public healthcare services were considered beneficial for providing essential care such as immunisations, antenatal care and family planning. Some participants appreciated the availability and functionality of these services, as reflected in statements such as:

‘The service are helpful for my child.’ (Person C, 14 years, Grade 7)

‘The support services provided at the clinics are very helpful to me and my baby.’ (Person H, 17 years, Grade 10)

However, others pointed out significant limitations, including a lack of youth-centred services and insufficient focus on teenage mothers’ needs. One participant noted:

‘The services should not only be for the child but for the mother as well.’ (Person H, 17 years, Grade 10)

Barriers such as school schedules, a lack of childcare during school hours and inadequate accommodations for school-going mothers were frequently cited. Participants explained:

‘If we can be accommodated as school attending people so that we do miss much at school because we are also want to pass school.’ (Person F, 18 years, Grade 11)

‘The services should not only be for the child but for the mother as well.’ (Person H, 17 years, Grade 10)

Some teenage mothers found the existing services helpful; the findings highlight the need for tailored, youth-friendly support systems that address both the practical and emotional needs of teenage mothers. Enhanced family, partner and healthcare provider support, coupled with more accommodating public health services, are essential for improving the experiences and outcomes of teenage mothers.

### Theme 3: Healthcare workers’ working ethics

The findings reveal significant challenges related to the attitudes and behaviours of healthcare workers, which greatly impact teenage mothers’ willingness and ability to access healthcare services. A positive, ethical approach from healthcare providers is essential for delivering high-quality care; however, participants reported various barriers stemming from judgemental and insensitive interactions.

#### Subtheme 3.1: Judgemental attitude and perceptions of healthcare workers

Teenage mothers expressed that judgemental attitudes and a lack of confidentiality from healthcare workers were significant deterrents to attending healthcare facilities. Many participants felt embarrassed and vulnerable because of the public nature of staff interactions and the lack of sensitivity to their specific needs. For instance, participants shared:

‘You are told everything in front of these people (referring to community members at the PHC facility) and the nurses are judgemental.’ (Person B, 16 years, Grade 9)

‘The nurses make it difficult for us to visit because they are always shouting at us, asking why we got pregnant at such a young age, and they do so in front of everyone – it is so embarrassing.’ (Person D, 15 years, Grade 8)

Others highlighted that staff often lacked understanding, with one stating:

‘The staff is old and has [*an*] attitude toward children; it would be better to have a younger person who understands the issues we face.’ (Person E, 17 years, Grade 11)

Such experiences created an environment where teenage mothers felt judged and unwelcome, ultimately discouraging them from seeking care.

#### Subtheme 3.2: Negative behaviour and practices of healthcare workers

The behaviour of healthcare workers further exacerbated these challenges. Participants reported instances of stigma, criticism, and a lack of respect, which negatively affected their willingness to engage with healthcare services. The participant recounted being publicly shamed by a healthcare worker:

‘This other day I went there for my baby in the morning wearing [*my*] school uniform. The old nurse called me a “whore” and said I should be at school learning.’ (Person B, 16 years, Grade 9)

‘… [*A*]t times because of the nurses that are always shouting us in front of the whole community it is embarrassing.’ (Person H, 17 years, Grade 10)

Others highlighted the lack of emotional sensitivity, with one stating:

‘At times, I want to ask about things I don’t understand, like how to get counselling because I am not okay, but the clinic staff only cares about the babies.’ (Person A, 16 years, Grade 9)

A recurring issue was the absence of confidentiality and privacy, with participants feeling exposed in front of other community members. As one participant explained:

‘There is no confidentiality at the clinic; we are shouted at in front of the whole community.’ (Person H, 17 years, Grade 10)

These experiences left many teenage mothers feeling unsupported, embarrassed and reluctant to return for further care.

The findings emphasise the critical need for training healthcare providers in youth-friendly, empathetic and respectful practices to address these barriers and foster an environment where teenage mothers feel safe and valued.

### Theme 4: Training and teaching

The findings highlight the critical need for improved training of healthcare workers and enhanced teaching for teenage mothers to address gaps in SRH education and support services. These aspects are essential for empowering teenage mothers and ensuring they receive holistic care.

#### Subtheme 4.1: Training of healthcare workers

Participants emphasised the importance of training healthcare providers to address the unique needs of teenage mothers with sensitivity and professionalism. Many teenage mothers reported negative experiences with healthcare workers, often because of judgemental attitudes and a lack of confidentiality, which deterred them from seeking services. As one participant noted:

‘Healthcare providers need to be trained on how to deal with teenagers.’ (Person B, 16 years, Grade 9)

Suggestions included improving staff attitudes, fostering a friendly and nonjudgemental environment and assigning younger nurses who might better understand the challenges faced by teenage mothers. Another participant stated:

‘Nurses can be less judgmental and more friendly … it will be easier to talk to them.’ (Person E, 17 years, Grade 11)

Building rapport and trust through training programmes focused on communication and empathy was identified as a key strategy for improving service delivery.

#### Subtheme 4.2: Teaching of teenage mothers

The lack of comprehensive education on SRH for teenage mothers emerged as a significant issue. Participants expressed that the healthcare system often focuses solely on the baby, neglecting the needs of the mother. They felt underserved in terms of guidance on contraception, prevention of infections and the importance of regular screenings such as Pap smears. One participant explained:

‘Healthcare providers only educate us on how to take care of the baby … there is no education provided about our health as mothers.’ (Person G, 19 years, left school in Grade 11)

Participants also highlighted the absence of SRH education in schools, which they attributed to cultural taboos and restrictive policies. This lack of information often leaves teenage mothers ill-equipped to make informed decisions. As one participant stated:

‘At school, there is no education regarding sexual and reproductive health … the issue of adolescent pregnancy is not addressed in our educational programmes.’ (Person H, 17 years, Grade 10)

They suggested that healthcare workers visit schools to provide education and advocate for including SRH topics in the curriculum to bridge this critical knowledge gap.

The findings underscore the need for dual efforts – training healthcare providers to deliver youth-friendly services and implementing targeted educational programmes for teenage mothers in both clinical and school settings. These initiatives are crucial to improving their well-being and equipping them with the knowledge and resources to make informed decisions about their health and futures.

## Discussion

The findings emphasise the need for more inclusive, confidential and accessible healthcare services tailored to the unique needs of teenage mothers, particularly those balancing school and caregiving or living in remote locations. Addressing these gaps is critical for enhancing the uptake of essential services and improving outcomes for teenage mothers and their children.

Gayatri et al.^19^ emphasise that teenage mothers’ access to public healthcare services is crucial for ensuring their safety and well-being, as well as that of their babies. In the community, teenage mothers actively use PHC services, particularly antenatal care, baby immunisation and family planning. This reflects their recognition of the value these services provide for their health and their children’s health. Immunisation is crucial for reducing morbidity and mortality, preventing school and work absences and rationalising healthcare expenditures.^20^

While PHC facilities offer many services, the findings indicate that teenage mothers tend to underutilise certain key services. Those who do not access SRH services face an elevated risk of unplanned pregnancies and STIs. Morris and Rushwan^21^ highlights the increased risk of exposure to STIs and unintended pregnancies because of an unmet need for family planning and a lack of access to SRH services. These gaps in service utilisation highlight the need for targeted interventions to ensure that teenage mothers fully benefit from the comprehensive care available to them.

Immunisation services are a consistent reason for teenage mothers to visit PHC facilities, demonstrating a responsible approach to ensuring their children’s health. The data indicate that many teenage mothers regularly have their babies immunised, reflecting their awareness of the importance of immunisations. Gayatri et al.^19^ emphasise that adolescent mothers frequently seek maternal healthcare services, including immunisation visits, as a key reason for engaging with PHC facilities. While PHC facilities offer a broad range of services, teenage mothers tend to underutilise several services, such as family planning, cervical smear screening, HIV testing and education on preventing sexually transmitted diseases. The data also reveal dissatisfaction among some participants with their treatment at healthcare facilities, contributing to a reluctance to seek services.

Underutilisation of PHC services among adolescent mothers significantly elevates the risk of adverse health outcomes, including maternal mortality.^22^ A systematic review focusing on low- and middle-income countries identified that adolescent mothers aged 15–19 years face higher risks of maternal morbidity and mortality compared to women aged 20–24 years, primarily because of their unique biological, sociological and economic circumstances. The study underlines that limited engagement with essential maternal health services contributes to these heightened risks.^23^ This emphasises the importance of enhancing access to and utilisation of PHC among teenage mothers to reduce the risk of maternal mortality rates.

Teenage mothers highlighted a significant gap in comprehensive SRH education in schools and healthcare facilities. The current focus is primarily on antenatal and postnatal care, with limited attention to broader aspects of sexual health. This lack of information leaves teenage mothers inadequately aware of the risks associated with early sexual activity and pregnancy.

In many communities, teenage pregnancy is considered a taboo topic. Participants found that discussions about SRH are often avoided at home, as parents fear such conversations might encourage sexual activity.^24^ This lack of guidance from family members contributes to teenage pregnancies and unmet family planning needs.^25^ These societal stigmas may also deter teenage mothers from seeking healthcare services because of a fear of judgement and stigmatisation.

Limited awareness of available healthcare services further deepens this issue. While some teenage mothers are familiar with services such as immunisations and family planning, many remain unaware of the full spectrum of healthcare offerings. This suggests a lack of proactive education by healthcare providers about the services available to them. Moreover, participants expressed dissatisfaction with the emphasis on infant care during postnatal visits, while their own health needs often go unaddressed. Govender et al.^7^ also observed that postnatal SRH education is frequently overlooked, which can lead to poor health decisions, such as repeated unintended pregnancies, STIs and mental health challenges.

Teenage mothers face significant challenges in accessing public healthcare services. Participants highlighted feelings of embarrassment when visiting healthcare facilities alongside elderly community members, as well as a lack of privacy and confidentiality. The exposure to the broader community fosters a fear of judgement and stigma associated with having a child at a young age and out of wedlock. The societal stigma surrounding adolescent sexuality and pregnancy can lead to negative labelling, social judgement and nondisclosure, deterring teenage mothers from seeking healthcare services.^25^ Ninsiima et al.^26^ reported that parents frequently hold negative perceptions of teenagers accessing family planning services, believing that adolescents under 18 years should not be sexually active and should instead focus on their education.

The inability of healthcare facilities to accommodate school-going teenage mothers presents another significant barrier. Timing conflicts often result in missed appointments, and some healthcare providers refuse to assist teenage mothers outside specified times. Participants shared that these challenges have led to school dropouts or academic failure. Gatsinzi^27^ emphasises that education is a basic human right, yet it remains inaccessible for many because of social disparities, such as teenage pregnancy. Addressing this issue requires collaborative efforts from educators, healthcare providers, parents, and public health officials to create supportive environments that promote both health and education for teenage girls.

Geographical and transportation barriers also hinder access to healthcare. Teenage mothers residing in remote areas expressed difficulty attending facilities because of long distances and limited transportation options. For those living on farms, transportation to town is infrequent and often dependent on the farmer’s schedule or the availability of mobile clinics. These logistical challenges further limit access to essential healthcare services, compounding the difficulties teenage mothers face in maintaining their health and that of their children.

Social support plays a critical role in the well-being of teenage mothers, influencing their ability to manage stress and navigate parenting challenges. Emotional, instrumental and affectionate support strengthens positive health outcomes, particularly during significant life transitions such as childbirth.^28^

Participants highlighted the importance of family support, particularly from mothers, in caring for the child and enabling them to attend school. Families help ensure the child’s developmental needs, including immunisations, are met, reducing the burden on teenage mothers. Family support also alleviates stress and improves emotional well-being.^29^ Partners, where present, contribute financially and emotionally, but many participants reported absent fathers, leaving the financial burden on families and social grants, which are often insufficient.^30^ A lack of family support sometimes forces teenage mothers to drop out of school or cohabit with partners to manage childcare responsibilities. Social support from family and partners is crucial for improving maternal mental health, enabling continued education and supporting child development.^29^

The findings further revealed mixed experiences with healthcare providers. Some participants appreciated the education and support, such as guidance on immunisations, baby care and accommodations for school-going mothers. However, many reported judgemental attitudes and sociocultural stigmas in healthcare settings, which hindered access to care. Nonjudgemental, supportive approaches are essential to improve health outcomes for teenage mothers and their children.^31^

Primary healthcare services, including antenatal care, immunisations and postnatal care, were valued by participants for promoting child health, such as protecting against diseases and addressing nutritional concerns. However, some participants felt these services focused solely on the child, neglecting the mother’s needs, and did not accommodate school-going teenage mothers, forcing difficult choices between education and healthcare.^7^ Poor access to healthcare services can result in delayed care, poor outcomes and repeated pregnancies. Tailored healthcare programmes for teenage mothers can improve infant care, education retention and reproductive health outcomes, representing an investment in national health and economic prosperity.^32^

Teenage mothers fear using healthcare services attributable to a lack of privacy and confidentiality. Participants reported that their issues were often addressed publicly, causing embarrassment, especially when elderly community members were present. Older healthcare providers were perceived as particularly judgemental, which discouraged the consistent use of SRH services. Stigma and discriminatory treatment further alienated teenage mothers, who felt neglected during visits.^25^

Healthcare workers’ negative attitudes and behaviour were identified as a major factor contributing to the poor utilisation of services.^4^ Providing youth-friendly, nonjudgemental services tailored to the needs of teenage mothers is crucial for improving their healthcare experiences and outcomes. Participants described instances of stigma, ill-treatment and a lack of respect, privacy and confidentiality. Healthcare providers often allowed their personal beliefs to influence their interactions, creating an environment that was unwelcoming and punitive for teenage mothers.^33^ These behaviours exacerbate feelings of isolation and marginalisation among teenage mothers, further deterring them from seeking care. Improving healthcare workers’ understanding and addressing these negative behaviours are essential steps in making public healthcare services accessible and supportive for teenage mothers.^4^

The findings highlight the need for improved training of healthcare workers and better education for teenage mothers to address gaps in SRH services. Enhanced training and education can help reduce stigma, improve attitudes and encourage greater use of healthcare services by teenage mothers, ultimately promoting healthier outcomes. Govender et al.^7^ suggest that training healthcare workers on adolescent health needs and understanding relevant laws and policies can enhance care delivery and reduce stigma. The South African National Adolescent-Friendly Clinic Initiative (NAFCI) is one such effort aimed at improving the quality of services for teenagers by providing healthcare workers with comprehensive training on adolescent needs. This initiative has proven effective in equipping healthcare workers with the knowledge to address teenage-specific health concerns, reducing judgement and stigma and fostering trust between healthcare providers and young mothers.^34^

The study also revealed that teenage mothers often lack knowledge about SRH because of limited education at home, in schools and at healthcare facilities. Participants noticed that cultural norms and parental reluctance to discuss sexual health contribute to this gap, as SRH education is often viewed as encouraging early sexual activity. This perspective is supported by research.^24,25^ Hako and Shipalanga^35^ emphasised that youth-friendly service initiatives should combine facility-based approaches with community engagement to promote SRH knowledge and psychological well-being among teenagers.

Teenage mothers expressed dissatisfaction with restricted SRH education in high schools, a limitation also observed by Govender et al.^7^ Participants suggested collaboration between schools and healthcare providers to deliver comprehensive SRH education, ensuring teenagers gain the skills and knowledge to make informed decisions about their health. Hako and Shipalanga^35^ further advocate for integrated efforts between schools and healthcare facilities to create awareness, enhance decision-making skills and promote healthy sexual behaviour among teenagers.

By addressing these gaps through training healthcare workers and integrating SRH education into schools and communities, the barriers to accessing care for teenage mothers can be reduced, fostering improved health outcomes for both mothers and their children.

## Recommendations and conclusion

The findings of this study emphasise the critical need for improvements in the accessibility, support, ethics and education in public healthcare services to address the unique needs of teenage mothers. The following recommendations are made:

To improve access to public healthcare services, promoting available services through platforms such as social media and schools is necessary. Collaboration between public healthcare services and school governing bodies is essential to deliver SRH education. Tailoring healthcare services to the needs of teenage mothers, including innovative solutions for school-going teenagers and strengthening mobile clinic availability, is crucial. Community healthcare workers (CHWs) should proactively trace teenage mothers who miss appointments and provide home-based assessments and counselling. Healthcare centres must adopt youth-friendly standards as per the National Adolescent and Youth Health Policy, ensuring operational adjustments such as flexible service hours to accommodate teenage mothers.

Support for teenage mothers can be enhanced by creating community-based support groups through CHWs. These groups should focus on counselling, health education, parenting skills and promoting school continuity and independence. Healthcare workers should be trained in adolescent-competent care to improve communication and better understand teenage development. Schools must ensure that pregnant teenagers remain in school and receive adequate support, while a combined approach involving schools, communities and health organisations can ensure effective family planning education.

Ethics awareness among healthcare workers is vital. Refresher workshops on the code of conduct and interpersonal skills training should be mandated to promote a nonjudgemental, youth-centred approach. Healthcare providers must respect privacy and confidentiality while fostering trust and openness with teenage mothers. Performance reviews should assess adherence to these principles to ensure accountability.

Training and teaching initiatives should focus on recruiting skilled healthcare workers committed to working with teenagers and involving parents in SRH education. Collaboration between the Departments of Health and Basic Education is necessary to incorporate sexual health education into school curriculums and to deploy school health nurses for regular student engagement.

Policy reforms should ensure the implementation of youth health standards in PHC facilities, emphasising interdepartmental collaboration to support teenage mothers. Educators should be equipped to refer teenage mothers to appropriate healthcare services, and policies should advocate for accessible SRH education in schools.

In conclusion, this study emphasises the critical need for tailored, youth-friendly healthcare services to address teenage mothers’ unique challenges in the Enoch Mgijima district in the Eastern Cape. Further research should explore healthcare workers’ perceptions of teenage pregnancy and their roles in supporting teenage mothers to deepen understanding and refine interventions.

### Limitations and handling of bias

It is acknowledged that the researcher’s professional background as a nurse could have influenced participants’ responses. However, multiple strategies were employed to minimise this risk, including an interview guide, triangulation and reflexive analysis. While the possibility of response bias because of the researcher’s professional background exists, the trustworthiness and rigour of the study were ensured through transparent methodology, ethical considerations and adherence to qualitative research principles.
